# Digital metamaterial filter for encoding information

**DOI:** 10.1038/s41598-020-60170-8

**Published:** 2020-02-24

**Authors:** Eistiak Ahamed, Mohammad Rashed Iqbal Faruque, Md. Jubaer Alam, Mohd Fais Bin Mansor, Mohammad Tariqul Islam

**Affiliations:** 1 Space Science Center (ANGKASA), Universiti Kebangsaan Malaysia, 43600 UKM Selangor, Malaysia; 2 Dept. of Electrical, Electronic & Systems Engineering, Universiti Kebangsan Malaysia, 43600 UKM Bangi Selangor, Malaysia

**Keywords:** Metamaterials, Characterization and analytical techniques

## Abstract

A new approach to controlling the flow of a plasmatic electron packet at the interface between metallic and dielectric layers is described. The proposed metamaterial structure operates in the optical frequency range and can be used as a digital processing filter. It exhibits two double negative resonances and one special passband region, while the existence of a metal-dielectric nano-tunnel enhances electromagnetic wave-metal interactions. The structural arrangement of this metamaterial coupled with the tunnel layer can effectively control the electric field and allows digital encoding of electron packets.

## Introduction

Metamaterials strongly affect the propagation of electromagnetic waves due to their special structural arrangements that cannot be found in nature. In particular, light-matter interactions in their nanostructure have been optimised in previous works to achieve non-typical electromagnetic responses. The latter includes the negative electric permittivity and magnetic permeability, which can help to bridge the gap between the fundamental nanoscience and nano-devices^1^. Owing to the artificial design of nanomaterials, they are widely used in electromagnetic devices including holographs, chiral plates, angle independent absorbers, and wave detectors^2^. Furthermore, the data transformation^3^ at large distances using integrated photonics is expected to play an increasingly important role in optical shielding^4^, imaging^5^, cloaking^6,7^, and sensing^8,9^ because of its potential ability to significantly reduce the costs and weights of these systems.

A new era in the field of information transfer has begun when coded, programmable, and digitalised metamaterial concepts started their gradual development on a larger scale^10^. Metamaterials used for digital applications possess various exotic functionalities, such as anomalous reflections, broadband diffusion, and polarisation conversion^11^. Previously, Giovampaola and Engheta^11^ utilised digital metamaterials consisting of negative metal and positive dielectric layers for manufacturing lens and tunnelling applications operated at optical frequencies to create metamaterial bits. Cui *et al*.^12^ developed a programmable, coded, and digital metamaterial to manipulate the reflections, scattering, and diffusion of waves in the microwave frequency range. Silveirinha and Engheta^13^ proposed a tunnelling structure, in which electromagnetic waves passed through a narrow channel filled with epsilon-near-zero materials. Shen *et al*.^14^ described a transmission-type coding metasurface whose transmitted field pattern depended on a particular meta-structure containing metamaterial bits. Gao *et al*.^2^ employed the digital coding of meta surfaces to control terahertz (THz) radiation. For this purpose, they developed a Minkowski closed-loop based coding unit that could generate multiple bits on different geometric scales.

In this study, a new metamaterial nano-structure is proposed for introducing digital filters that can perform bit sequence conversion in the frequency range of 200–600 THz. This nano-structure exhibits two distinct resonances in the negative electrical permittivity and negative magnetic permeability regions. It is also used to produce a special tunnelling arrangement whose performance is superior to that of a regular metamaterial array structure. A normal digital unit cell introduced in this work contains a dielectric substrate used as a bit element (called ‘0 element’) and a metal bit element (called ‘1 element’). By designing appropriate tunnel sequences, it is possible to successfully manipulate the THz range and digitally convert propagating waves through digital metamaterial filters for encoding electromagnetic signals.

### Realizing the digital filter

**Figure 1:**
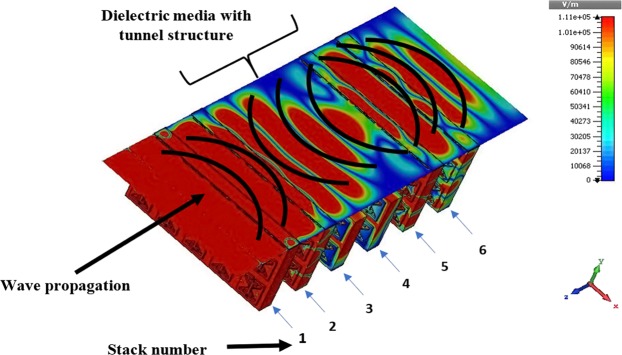
Backward wave propagation in tunnel metamaterial arrangement.

The basic design block can contain 5 × 5 array pattern in which the central unit cell is surrounded by two identical unit cells. Limited pattern combination is used to minimize the size of the structure. The unit cell and array pattern are designed in Figs. S1 and S2, respectively. In array pattern (Fig. S2), the unit cell is indicated with *i*, and surrounding unit cells are denoted as *i* + 1 and *i* + 2. These two-unit cells present a minimum array pattern that can obtain better responses for tunnelling arrangement. The structure is tested by backward wave propagation through the metamaterial array device in TE (Transverse Electric) condition. The designed array pattern is more sophisticated and efficient when the six array stacks are used in tunnel structure and significantly increases electric field contour intensity. Figure 1 exhibits the backward wave propagation at 215 THz. This full design system has six stacks and tunneling stacks numbered as 3^rd^ and 4^th^. First two stacks are manipulating wave in forward propagating direction but after that, the dielectric media with tunnel stacks (3^rd^ and 4^th^ stacks) exhibits backward wave propagation and in last two stacks (5^th^ and 6^th^) the wave again propagate in forward direction. Based on that array propagating characteristics, the metamaterial filter device pattern is achieved.

**Figure 2:**
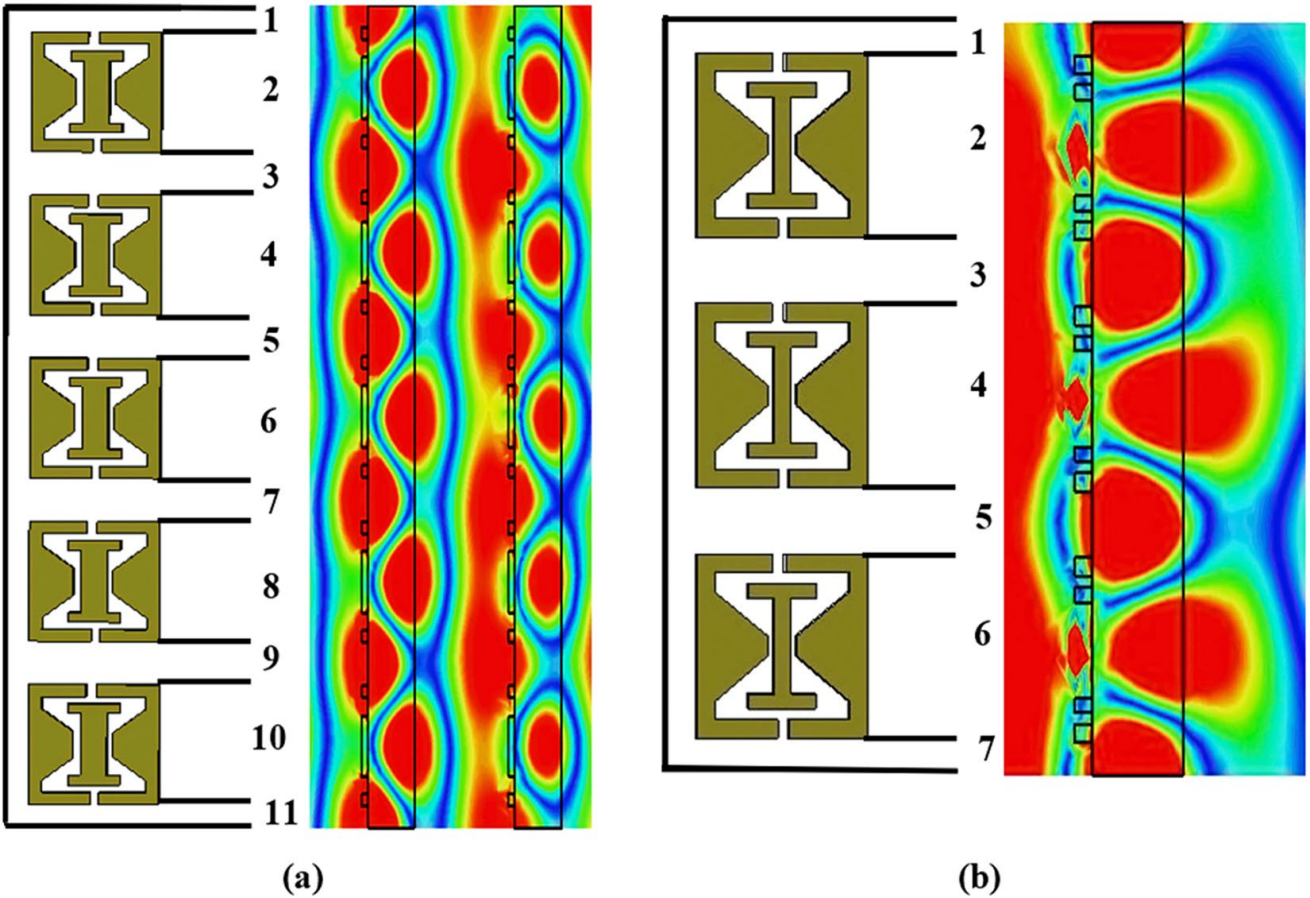
Intense electric clouds produced by the metal and dielectric parts (**a**) 5-unit cell (**b**) 3-unit cell.

The array structures have five-unit cells and that can be divided into dielectric and metal elements. Therefore, the five-array structure creates 11 electron clouds, as shown in Fig. 2(a). The proposed array pattern consists of five-unit cells; therefore, the dielectric and metal parts create eleven intense electron clouds. The clouds number can be identified based on the condition *(i* × *2) +* 1 where, *i* is the unit cell number. When array pattern has three-unit cells, it creates seven clouds shown in Fig. 2(b).

## Tunnel Structure of the Proposed Nano-Metamaterial Device with Equivalent Circuit Model

**Figure 3:**
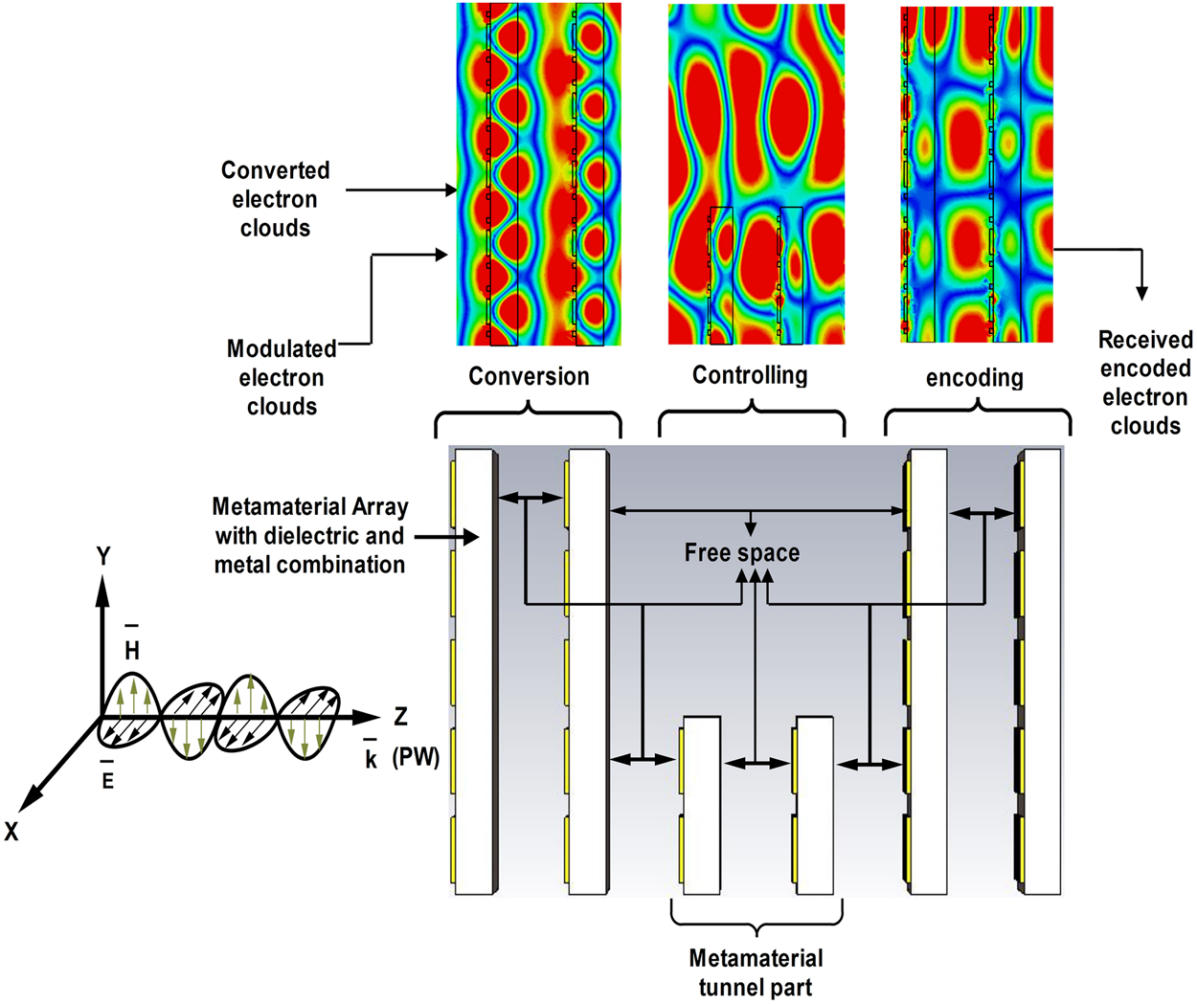
A proposed tunnel structure and its operation.

Optical information processing through a filter is a hot research topic^15^. Optical communication data sets can be encoded by their amplitude, phase, intensity, wavelength or polarisation. Therefore, a unit cell is used (see in supplementary file) to create an array whose characteristics match those of the unit cell. The obtained array plate is further arranged in parallel sequences to achieve desired properties of the proposed nano-structured metamaterial. Furthermore, two different array structures, in which one arrangement exhibits tunnelling properties, and the other provides a gap in the tunnel space have been designed as well. The resulting tunnel structure is illustrated in Fig. 3 where the entire metamaterial arrangement is divided into three parts corresponding to the conversion, controlling, and encoding processes. These parts react with a propagating electromagnetic wave as shown in the figure.

**Figure 4:**
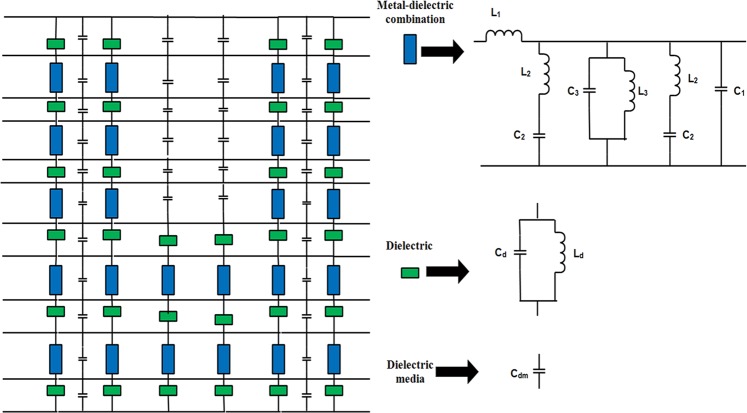
Equivalent circuit and its symbolic conversion.

The equivalent circuit model for digital tunnel filter is exhibited in Fig. 4. It characterizes a unit cell equivalent circuit that can be named as metal-dielectric combination (blue block), where *L*_1_ represents as self-inductance of unit cell and *C*_*1*_ termed as self-capacitance. The *C*_2_ and *L*_2_ are presented for the E shape effect on unit cell, whereas, *C*_3_ and *L*_3_ for the I shape effect for equivalent circuit; besides, only dielectric part is presented with the green block, which has parallel capacitance *C*_*d*_ and inductance *L*_*d*_. The dielectric media (free space) termed as only capacitor *C*_*dm*_. The circuit is designed based on LC mode in SRR structure^16^. According to LC mode of SRR structure^2^ the kinetic (L_k_) and Faraday inductance (L_F_) of any SRR structure depend on its metal and dielectric combination. The inductance and capacitance can be estimated based on the Eqs. 1–2^17,18^.

1 $$L(nH)=2\times 10-4\,l[ln(\frac{l}{w+t})+1.193+0.2235(\frac{w+t}{l})]$$

2 $$C(pF)={\varepsilon }_{0}{\varepsilon }_{e}F(k)$$

$${\varepsilon }_{e}=1+\frac{({\varepsilon }_{r}-1)F(k)}{F(k1)}$$

where *ε*_*e*_, *ε*_*r*_ and *ε*_0_ are the effective dielectric constant, relative permittivity and free space permittivity, *F*(*k*) is the ratio between the complete elliptic integral of the first solution *K*(*k*) and h is the substrate thickness.

**Figure 5:**
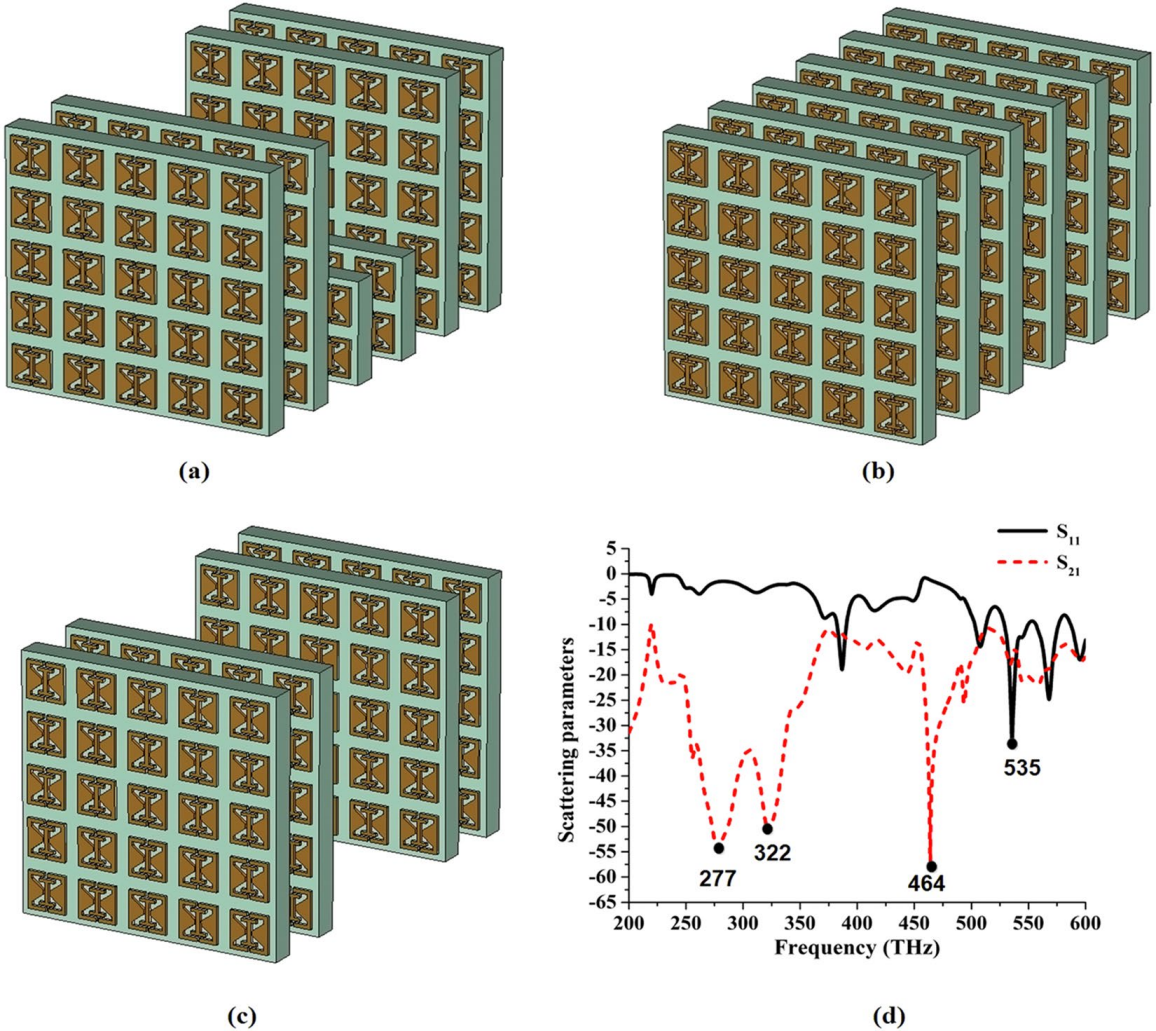
(**a**) A proposed tunnel metamaterial array structure, (**b**) full array metamaterial, and (**c**) tunnel gap. (**d**) Scattering parameters of the prepared tunnelling structure.

The proposed designed tunnel structure and other two structures are depicted in Fig. 5(a)–(c), while tunnel scattering parameters are described in Fig. 5(d). The first structure (Fig. 5(a)) exhibits resonances at 277, 322 and 464 THz, while it also shows a stop band frequency at 535 THz; the full array (Fig. 5(b)) and structure with a tunnel gap (Fig. 5(c)) resonate at 280, 325, and 460 THz and at 275, 340 and 456 THz respectively, while they exhibit stop band resonances at 538 and 558 THz respectively; and the unit cell shows resonances at 306 and 366 THz, while it exhibits passband frequency at 534 THz. The scattering parameters of the tunnel structure are better than those of the unit cell and one plate array. In addition, the properties of the proposed tunnel structure are determined by analysing the propagation of TEM waves, which partially interact with the first array plate and become converted into eleven electron clouds then controlled by the tunnel part. The encoding part encodes the controlled electron clouds and sends the generated signal to the receiving part. Waveguide port 1 transmits the modulated electromagnetic electron clouds, and port 2 receives them. The proposed dielectric-metal interface has two parts that can be employed by binary 1(metal) and 0 (dielectric). The electromagnetic fields are principally confined within the metal-dielectric interface with the evanescent decay ${e}^{-{k}_{z}z}$ inside the metal, where *k*_*z*_ is the transverse component of the wave vector (z-direction). Therefore, after facing electromagnetic wave, the interface creates evanescent decaying and help to convert the information into electron clouds. The phase of electric field is changed after π/2 degree later, therefore, the input and output responses are also changed according to the e-field phase response. The electron has a special capability to convey the information from input to output portion.

**Figure 6:**
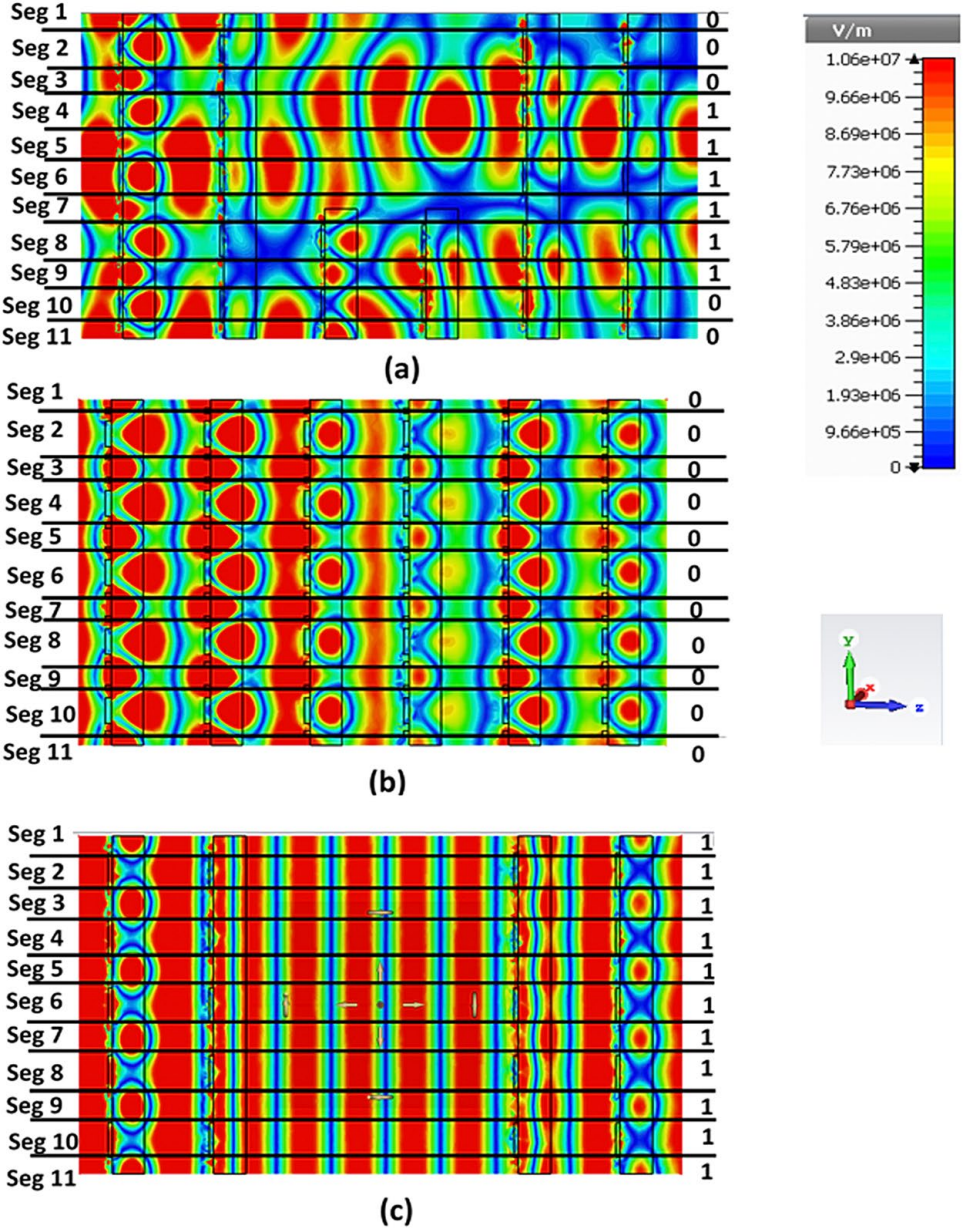
Electric field distributions obtained for the (**a**) tunnel structure, (**b**) full array structure, and (**c**) tunnel gap.

The intensity of the induced electric field is controlled by the tunnelling structure and not by other two structures. By changing the structural arrangement, the intensity of the propagating TEM wave can be varied while adjusting the electric filed intensity. The electron clouds created by the TEM wave propagation in the tunnel structure are used to convert the electrical signal from information, when propagation wave (PW) hit the 1^st^ array structure and collect from the last metamaterial slab. Therefore, the entire structure can be divided into 11 segments depending on the metal and dielectric layers of the metamaterial tunnel arrangement. First, the propagating electromagnetic wave is incident on the first metamaterial array, and the modulated electron clouds are divided segment wise. These clouds are converted by the insertion of the tunnel (Fig. 6) that can be explained digitally. In general, the free electron movement at a dielectric-metal interface can be used for signal encoding in terms of voltage or current. In this work, information is digitised based on voltage. The red area indicates high voltage denoted by ‘1’, while the other coolers are digitally low and labelled by ‘0’. In Fig. 6(a), the segment 1 indicates that the electron cloud is digitally low which results in a low conversion point. In segments 2, 3, 10 and 11, the converted clouds are also digitally low as indicated by the ‘0’ symbol. In segments 4, 5, 6, 7, 8, and 9, the electron clouds are digitally high. This phenomenon is observed for the tunnel structure; however, the other two structures are also analysis in same phase and same intensity, but their digital responses do not change (at same phase they provide low and high states) (see Fig. 6(b),(c)).

**Figure 7:**
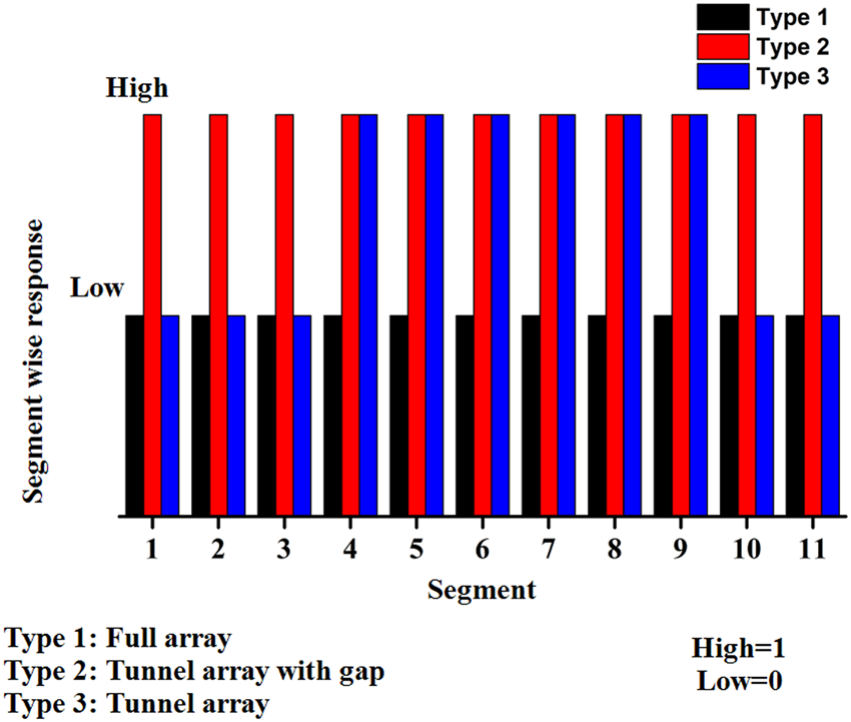
Digital responses of the proposed tunnel structure and two other array structures.

Figure 7 shows the digital responses obtained for different segments of the three structures. Here, the electric clouds are controlled by the tunnel structure, and the other two structures are used for encoding information. In this figure, types 1 and 2 represent the full array structure and tunnel array with a gap, respectively, while type 3 corresponds to the tunnel array. These results indicate that digital information responses are high (‘1’) in segments 4, 5, 7, 8, and 9 and low (‘0’) in the remaining segments (in case of tunnel structure).

### Effect of dielectric media on tunneling area

**Figure 8:**
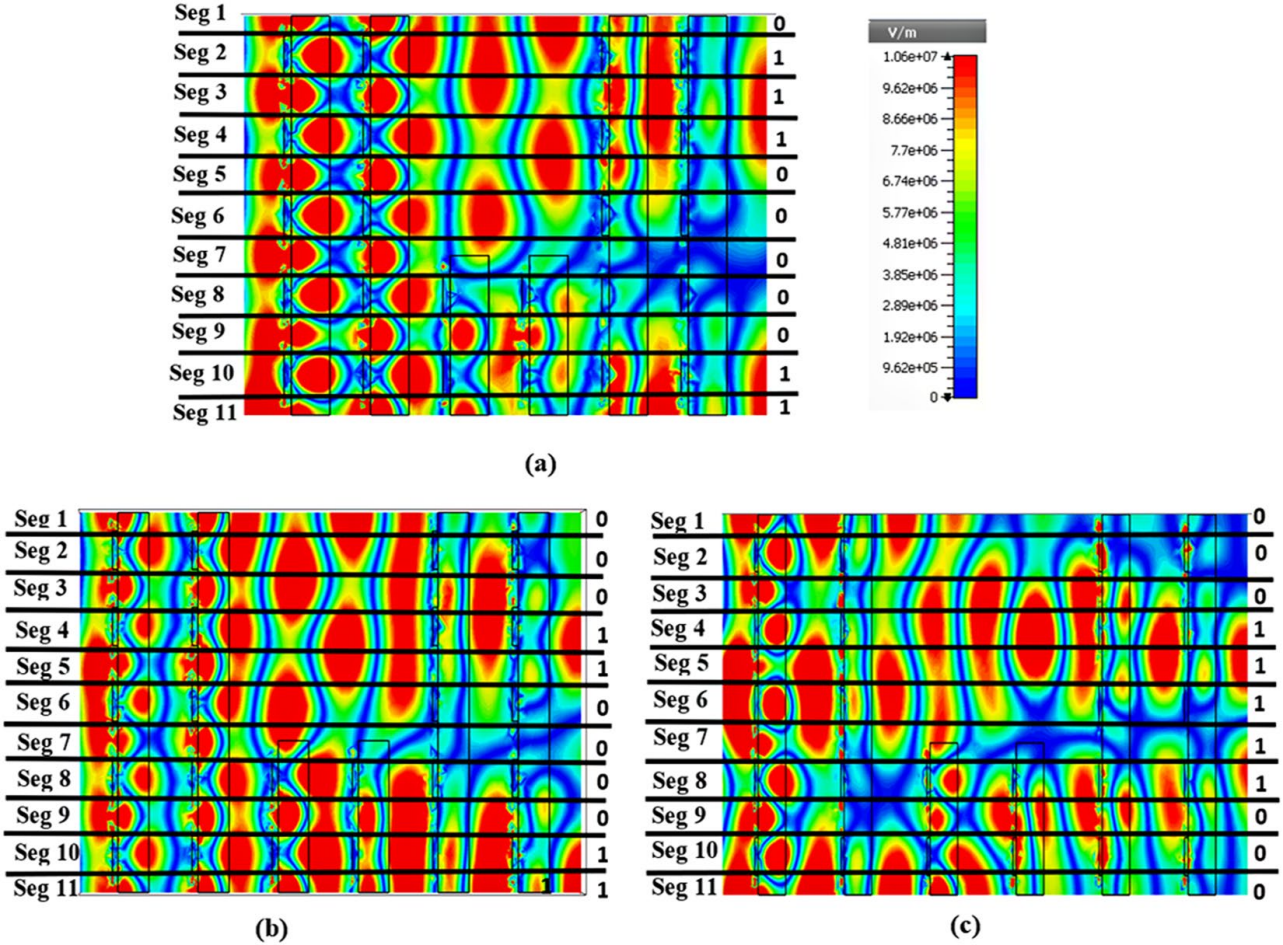
The dielectric media (free space) variation between array stacks. From one stack to another stack distance (**a**) 170 nm (**b**) 255 nm and (**c**) 340 nm.

**Figure 9:**
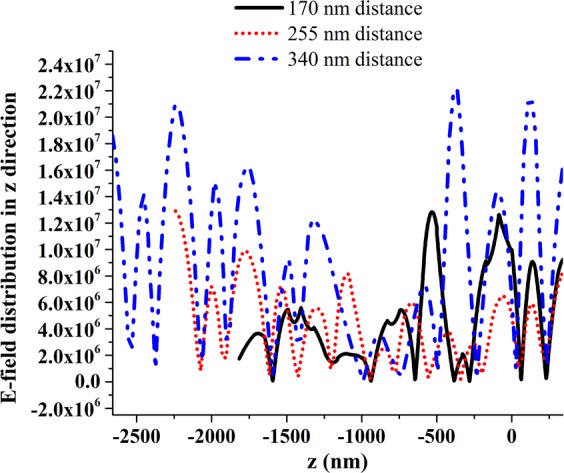
Induced e-field responses along wave propagation direction for 170 nm, 255 nm and 340 nm.

In tunnel configuration, the free space gap is termed as dielectric media which has a significant effect on wave propagation. Two types of effect can be explained; (a) by varying overall distances between all array stacks and (b) by varying the conversion and encoded part distance with fixed tunnel structure. Figure 8 briefly discusses about dielectric media variation effect on wave propagation in tunnel structure. First analytical effect is explained by maintaining distances among array stacks in 170 nm, 255 nm and 340 nm. In 170 nm array stack distance is the manipulated wave created 01110000011 binary pattern (see in Fig. 8(a)), besides, in 250 nm distance between array stacks is presented 00011000011 binary pattern (see in Fig. 8(b)) and in unit cell distance (340 nm) the device creates 00011111000 binary response (see in Fig. 8(c)). Among that three-metamaterial device, 340 nm distance-based structure induced strong electric field response in wave propagation direction which is exhibited in Fig. 9.

**Figure 10:**
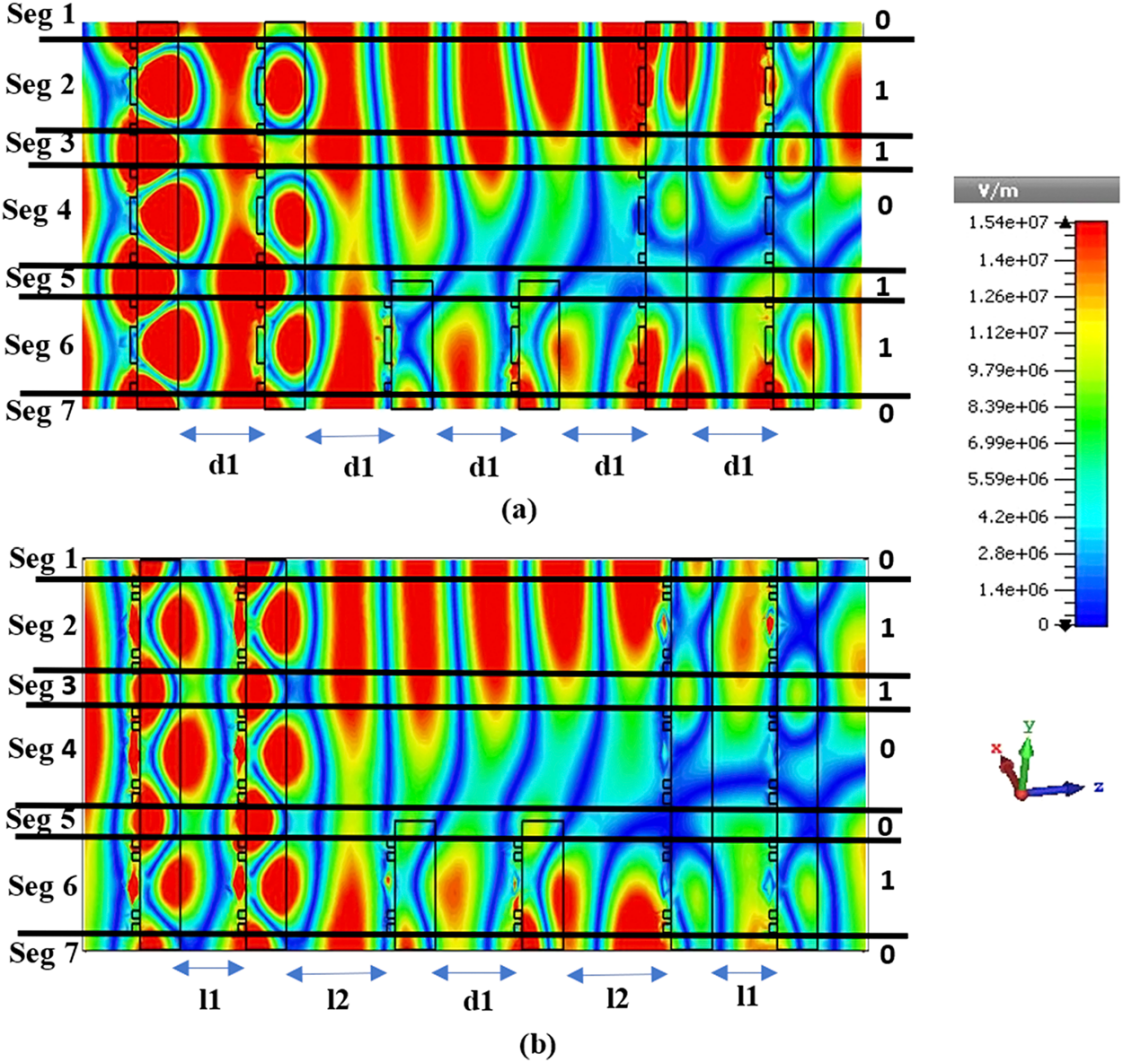
The tunnel array (3 × 3) pattern for digital responses. (**a**) constant distance d1 (340 nm) maintains in this tunnel array device and (**b**) larger dielectric media creates in tunnel area by shifting stack 2 and stack 5 where l1 is 255 nm, l2 425 nm and d1 340 nm. TEM wave propagation direction is in z axis, whereas, electric and magnetic field are applied in x and y axis, respectively.

In second dielctric media variation, 3 × 3 metamaterial array presents same structure as 5 × 5 tunnel array pattern where tunnel convey 1 × 3 array pattern. It produces 7 electron clouds (according to (*i* × 2) +1)) (see in Fig. 10). Due to the tunnel pattern and the fixed stack distance, the propagating wave faces an evanescent field effect and creates a binary pattern as 0110110 (Fig. 10(a)). After that, 2^nd^ and 5^th^ array plates are moved closer to stack 1 and stack 6 where it creates 0110010 binary pattern and exhibited in Fig. 10(b). Both responses are in same e-field intensity (indicated by colour bar) and exhibited in Fig. 10.

### Comparative validation of proposed tunnel model

**Figure 11:**
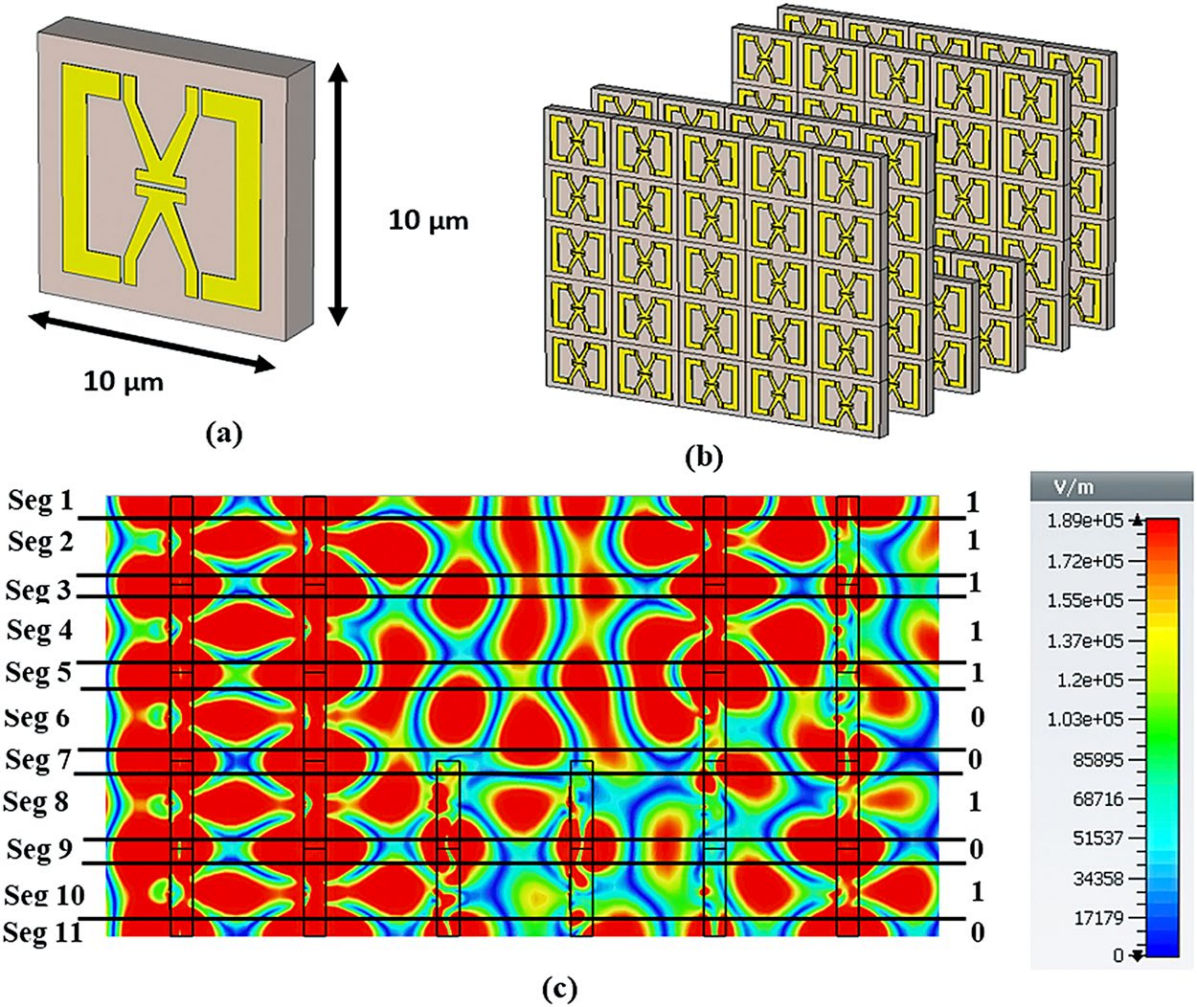
The new design for verification of the method. (**a**) V-C shape metamaterial (size 10 × 10 (μm)), (**b**) prepared tunnel array and (**c**) its e-field response at 29 THz with 11111001010 patterns.

The tunnel model has been redesigned for software-based result validation and is presented in Supplementary Fig. S8. The HFSS software is used for validation. For more verification, another C-V shape metamaterial is designed that creates a stop band frequency resonance in 29 THz. The designed metamaterial structure size is 10 × 10 μm^2^ (presented in Fig. 11(a)). Based on that unit cell, another tunnel structure model is designed and exhibited in Fig. 11(b). After applying same condition as applied for proposed tunnel design, the C-V tunnel structure gives binary 11111001010 responses in its output (see in Fig. 11(c)).

## Conclusion

A new metamaterial nano-structure is developed for constructing a digital information filter. The obtained configuration is fully controllable by the tunnel arrangement of the metamaterial structure, and the occurred processes are described for the optical frequency region. The electric field is mainly affected by the tunnel structure, and modulated information is encoded using the proposed meta-device. Furthermore, the resulting metamaterial tunnel arrangement can significantly enhance its Q-factor. The entire encoding process is explained numerically for one optical passband resonance frequency corresponding to the tunnel metamaterial device.

## Supplementary information

Supplementary information .
