# Supplementary material for: Digital metamaterial filter for encoding information
Source: Sci Rep. 2020 Feb 24;10:3289. doi: 10.1038/s41598-020-60170-8 (PMC7039869; doi:10.1038/s41598-020-60170-8)
Supplement: Supplementary file 1 — Supplementary information . [file 41598_2020_60170_MOESM1_ESM.docx]

**Digital metamaterial filter for encoding information**

Eistiak Ahamed^1^, Mohammad Rashed Iqbal Faruque ^1^, Md. Jubaer Alam^1^, Mohd Fais Bin Mansor^2^, Mohammad Tariqul Islam^2^

^1^Space Science Center (ANGKASA), Universiti Kebangsaan Malaysia, 43600 UKM, Selangor, Malaysia

^2^Centre of Advanced Electronic and Communication Engineering, Universiti Kebangsan Malaysia, 43600 UKM, Bangi, Selangor, Malaysia

eistiak.ahamed@gmail.com, rashed@ukm.edu.my, jubaer.alam@iubat.edu, m.mansor@ukm.edu.my

Corresponding author: rashed@ukm.edu.my

**Supplementary note 1:**

**Parameters of the unit cell:**

The geometry of the metamaterial structure is shown in Figure S1(a). Its unit cell is composed of corrugated metal strips labelled E−I in the alphabetical order and periodically arrayed in the x- and y-directions. The middle arm of the E part is filled with metal and has the shape of a prism for better structural stability. The two E strips mirror each other, while strip I is fixed. The proposed array structure is presented in Figure S1(b). It has a loss free quartz substrate with a dielectric constant of 3.75. Gold (Johnson)(optical) is utilised as a patch metal on the loss free quartz substrate with an ultimate thickness of 30 nm. The main reason for the success of gold nano-particles in the field of nanoscience and nanotechnology is the uniqueness of their optical properties related to the formation of surface plasmon polaritons. In particular, gold metal has a large number of polarisable conduction electrons, which is a general prerequisite for their preferential interactions with electromagnetic field and nonlinear optical phenomena.

The geometrical parameters of the unit cell are listed in Figure S1(a) and Table S1. The symbols ‘*a*’ and ‘*b*’ denote the substrate length and width, respectively. Furthermore, ‘*l*’ indicates the length of the *E* part, *d* is the width of the two legs of the *E* part, *e* is the gap between the middle and side arms of the *E* part, t is the length of the *I* part, *g* is the gap between the two inverse *E* parts, *w1* is the width of the *I* part, *z* denotes one side of the prism arm of the *E* part, *p* is the length of the prism arm, *s* marks the prism arm head, and *g2* is the distance between the metal arm and the substrate edge.

**Table S1:** Geometric parameters of the proposed metamaterial nanostructure.

| Parameters | Dimentions (nm) | Parameters | Dimentions (nm) |
| --- | --- | --- | --- |
| *a* | 340 | *g* | 20 |
| *b* | 340 | *w1* | 100 |
| *l* | 250 | *z* | 98.41 |
| *d* | 25 | *p* | 100 |
| *e* | 24 | *s* | 28 |
| *t* | 130 | *g2* | 45 |

(b)

(a)


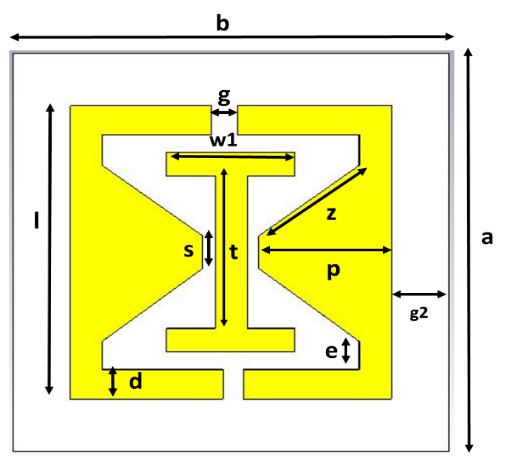

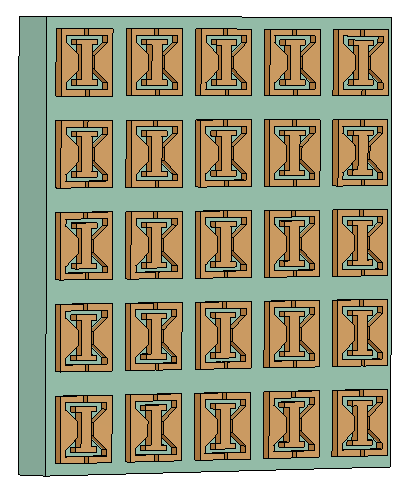


**Figure S1:**  Proposed metamaterial (a) unit cell and (b) array prototypes.

**Unit cell (*i*)**

**Unit cell length (*l*)**

***i+1*, *i+2***

**array**

**Figure S2:** Array and tunnel pattern design.

**Electromagnetic properties of metamaterial nanostructure:**

The proposed structure is analysed by the CST Microwave studio software, which represents an electromagnetic simulator based on a finite integration technique (FIT). In this model, the E−I structure is placed between the two wave ports to evaluate its scattering parameters under the ordinary boundary conditions. The latter correspond to the electric field placed along the x-axis, the magnetic field placed along the y-axis, and the electromagnetic field propagating along the z direction. The resultant scattering parameters (including the transmission and refraction coefficients) are extracted using the Robust method [S1]. The quasi-transverse electromagnetic (TEM) mode of wave propagation is applied in metamaterial simulations, which are performed in the frequency range between 200 to 600 THz. Using the Robust method, complex S parameters directly related to the refractive index n and impedance z are extracted via equations (1−10) that are shown in block diagram in Figure S3.

The reflection and transmission coefficients depend on the position of the first boundary (wave port) and thickness of the metamaterial slab, respectively. The proposed metamaterial is considered a passive medium because of its field dependability. The real part of the impedance and imaginary part of the refractive index are described by equations (3−4). Depending on that condition impedance can be determined by the equation 5. The refractive index can be calculated by the equation 7, where the real part of the refractive index n is the branches of the logarithm function.

Data extraction and process

Passive medium conditions

Effective parameter extraction

**Figure S3:** Effective parameters extraction process.

Using these parameters, the impedance is determined via equation (5), and the refractive index can be calculated by equation (7). Here, the real part of the refractive index n is an argument of the logarithm function, m is the integer value, $k_{0}$ is the wave vector in the free space, and d is the prototype thickness.

The proposed metamaterial nanostructure is characterised by a unique shape. Its metamaterial unit cell exhibits resonance frequencies at 335 and 386 THz, while it shows passband maximum point at 534 THz (see Figure S3(a)). The latter is further used to build a 5×5 array prototype whose resonance frequencies are close to the unit cell scattering parameters. After analysing its effective parameters, the proposed nano-structure can be considered a metamaterial (Figure S4(b)−(e)). Because the extracted parameters satisfy equations (3) and (4), the metamaterial represents a passive medium in the nano-structural range. The proposed metamaterial represents a left-handed system that simultaneously exhibits negative values of the effective permittivity, effective permeability, and refractive index in certain regions of the operating frequency bandwidth as shown in Figures. S4(c), (d), and (e).

(a)

(b)

(c)

(d)

(e)


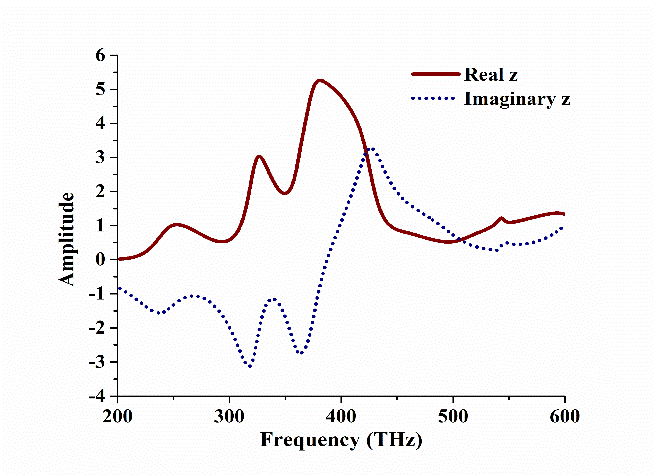

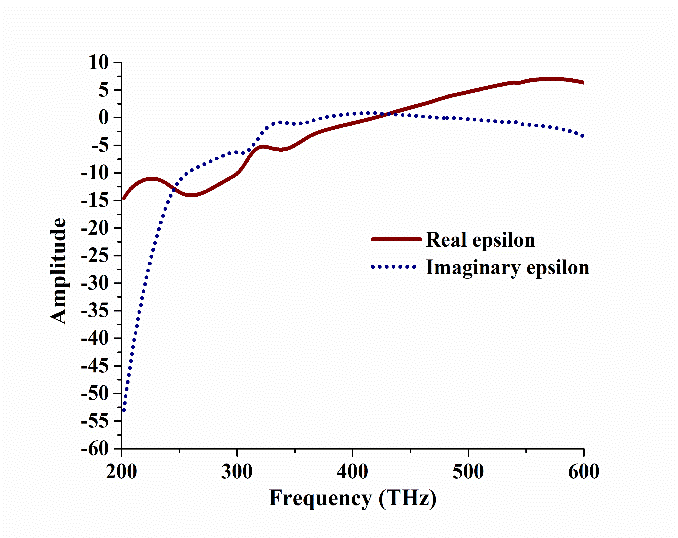

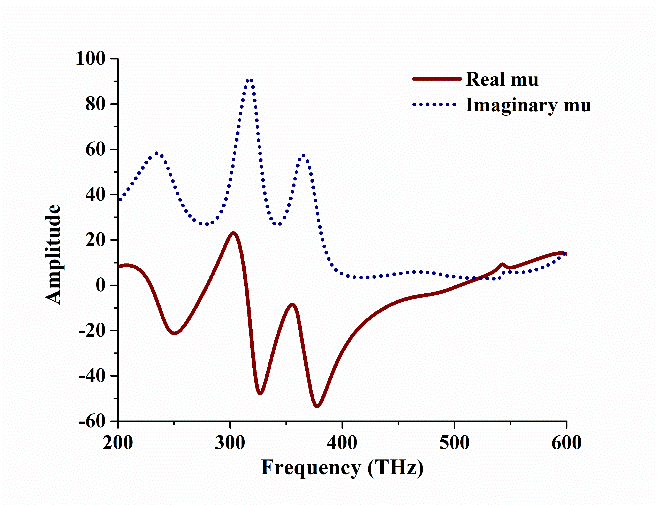

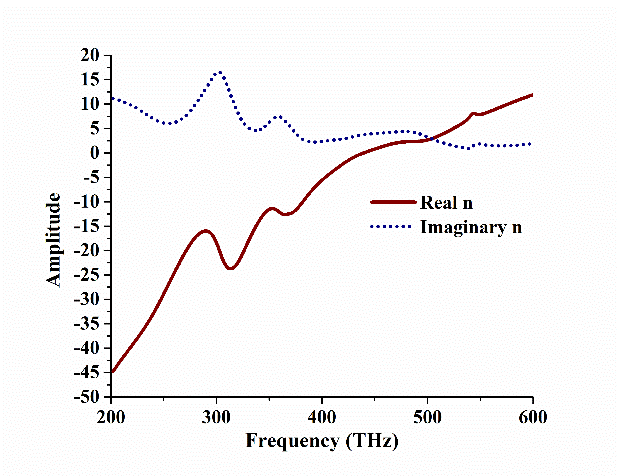

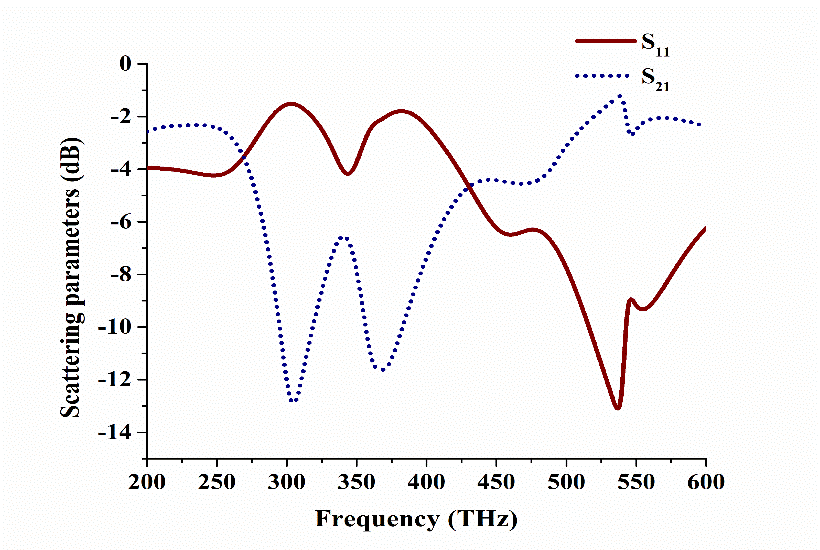


**306**

**366**

**534**

**Figure S4:** (a) Scattering parameters shows resonances at 306 and 366 THz (passband pick at 534 THz), (b) impedance z, (c) effective permittivity $ɛ$, (d) effective permeability $\mu$, and (e) refractive index n of the proposed nano-structure.

Figure S5 describes the metal-dielectric interaction and applied electromagnetic field along with the material surface. According to Figure S5(a), the frequency of the electric field is 534 THz (its intensity is high in the region indicated by the red colour). The frequency of the magnetic field depicted in Figure S5(b) is 534 THz. Figures S5(a) and (b) show that these two fields are generated in different places. The maximum surface current density is proportional to magnetic field response, therefore, on that region the surface current intensities are also maximum.


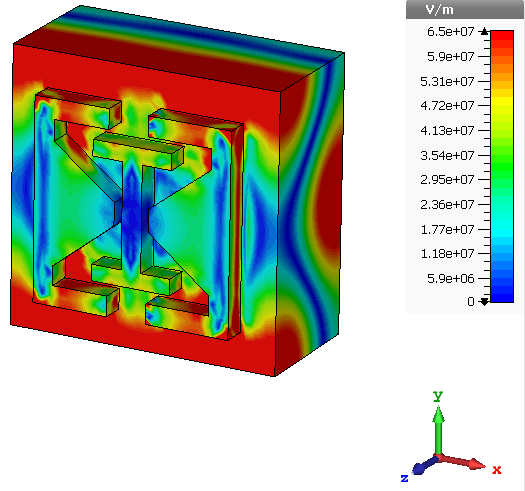


(a)


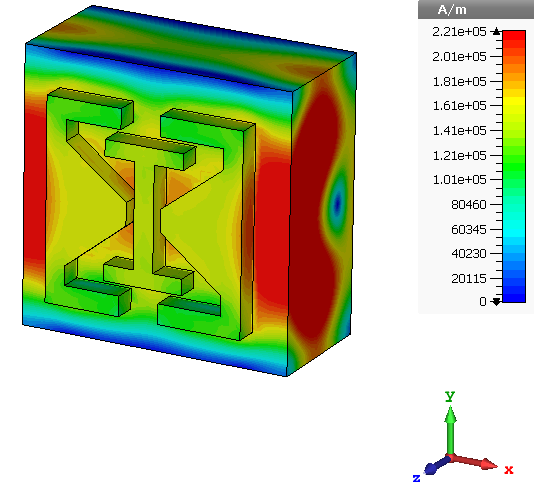


(b)

**Figure S5:** (a) Electric and (b) magnetic fields induced at passband frequency of 534 THz.

Figures S6(a) and (b) show the intensities of the electric and magnetic fields induced along the substrate x-axis direction in the proposed metamaterial structure at 534 THz. The amplitudes of the electric filed in the positive and negative x-directions are much higher than that of the electric field induced in the middle region of the substrate at the lower optical resonance frequency. In contrast, at the higher frequency, the amplitude of the electric field generated in the substrate is very different: it gradually increases in the middle region and decreases at the ends. The intensity of the induced field reaches minimum in the middle arm I, as shown in Figure S6(a). However, its magnitude becomes lower because of the capacitance effect of the split gap produced in both cases, and the skin depth is higher at the larger optical resonance frequency. Figure S6(b) shows the magnetic field intensities induced along x-axis.


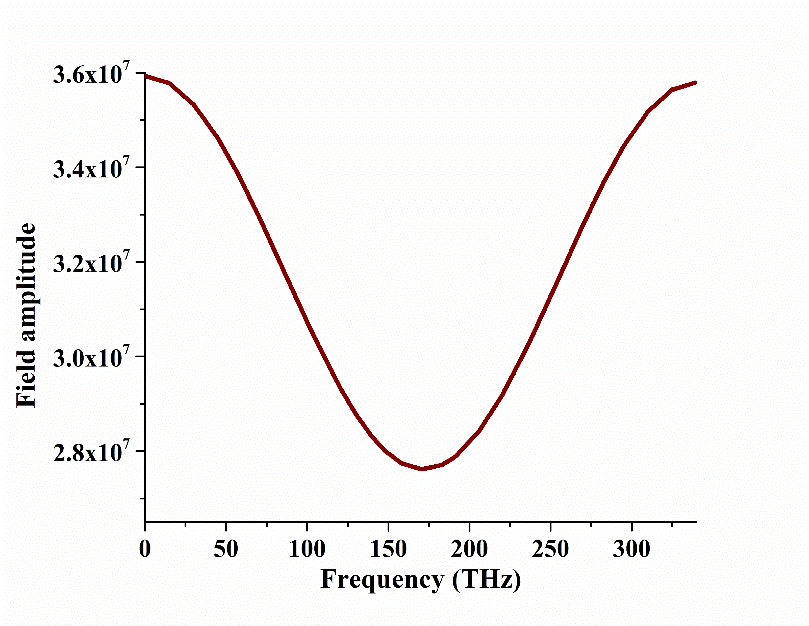

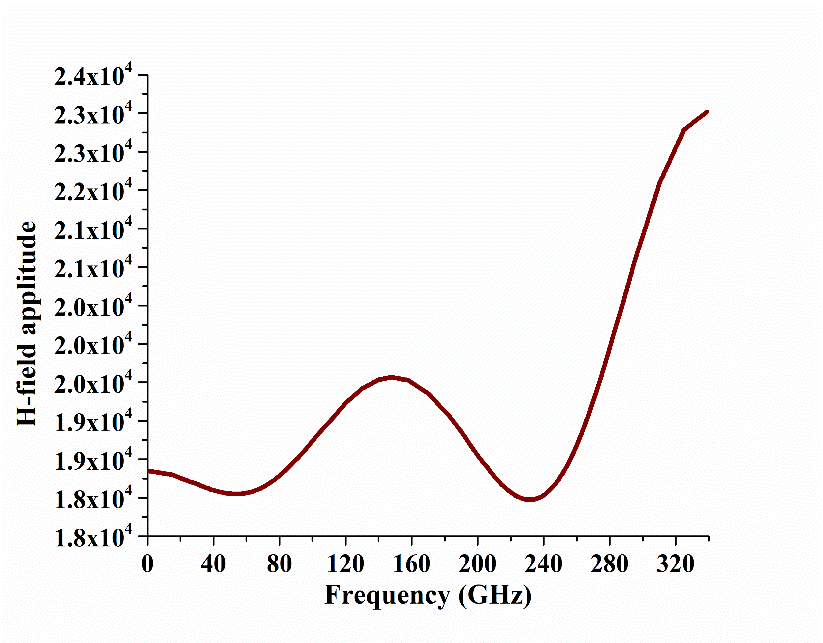


(b)

(a)

**Figure S6:** Amplitudes of the (a) electric and (b) magnetic fields measured along the x-axis at a first electric resonance frequency of 542.36 THz and magnetic resonance frequency of 567.88 THz.

**Electric field response in different phase**

The achieved electric field response for tunnelling model are taken at a same phase and same intensity (electric field) which is indicated by colour bar in Figure S7. The field is time harmonic, therefore, in first half cycle after checking the output response in next half cycle, the output of Fig. 3b was high (red) and the output of Fig. 3c was low (blue) that were totally opposite of previous half cycle. The taken responses are visualised below. Figure 1(a) (full array structure) and Figure 1(b) (tunnel gap array structure) are taken at 0 degree phase condition and Figure 1(c) (full array structure) and (d) (tunnel gap array structure) are taken at 90 degree phase, both of the cases the e-field intensity is 1.06 e + 07 v/m.


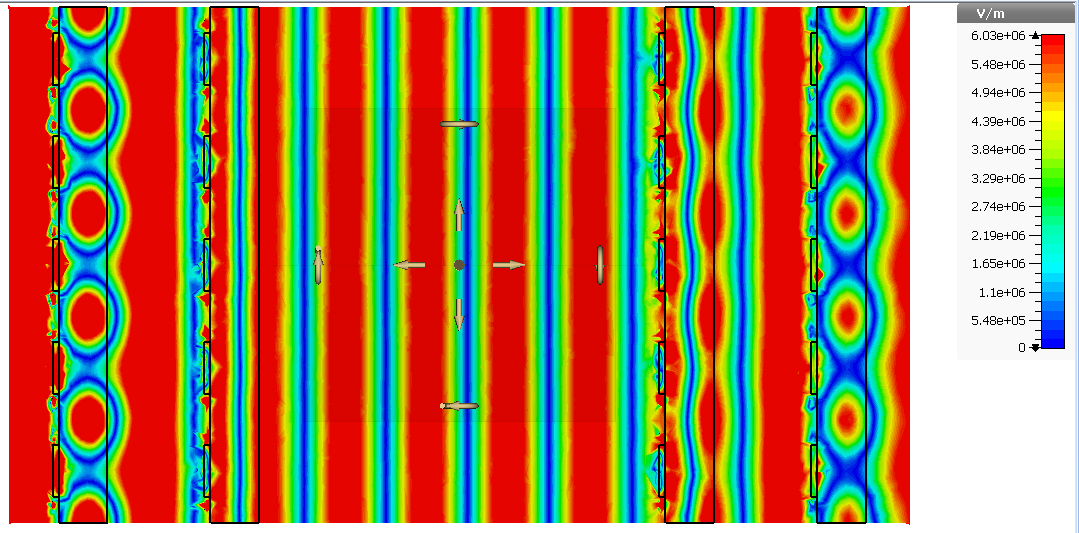

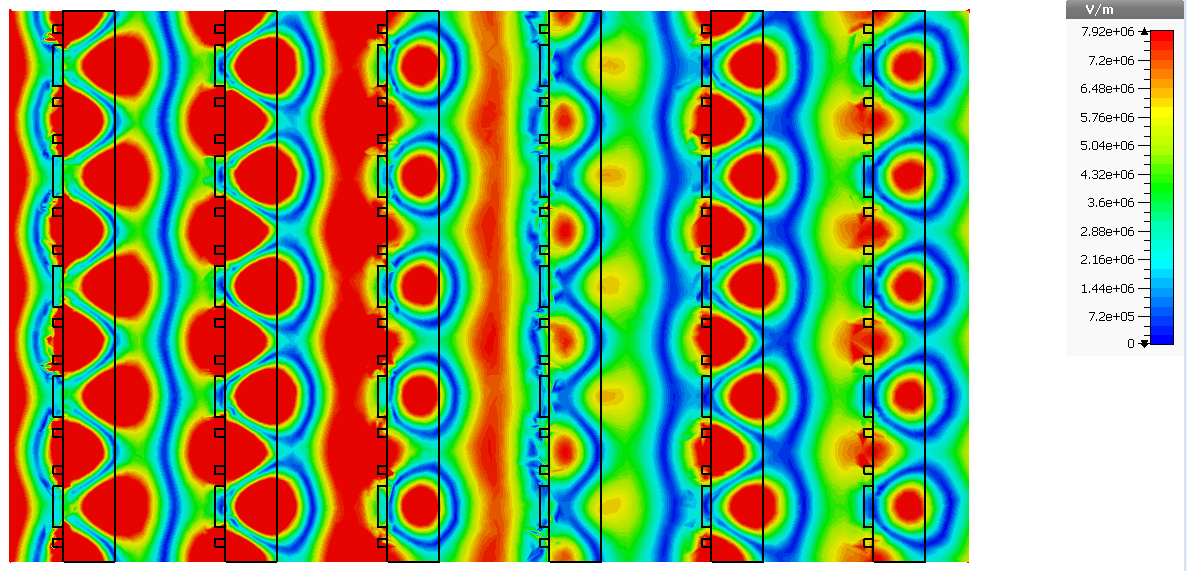

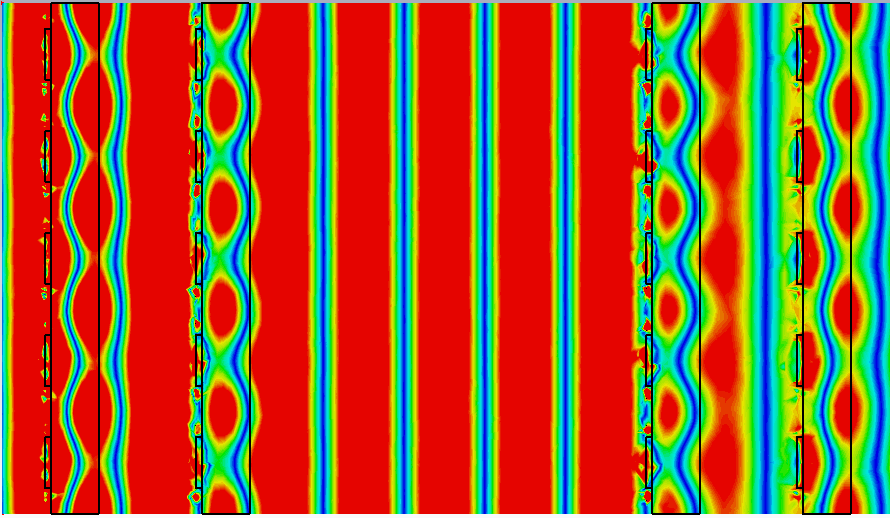

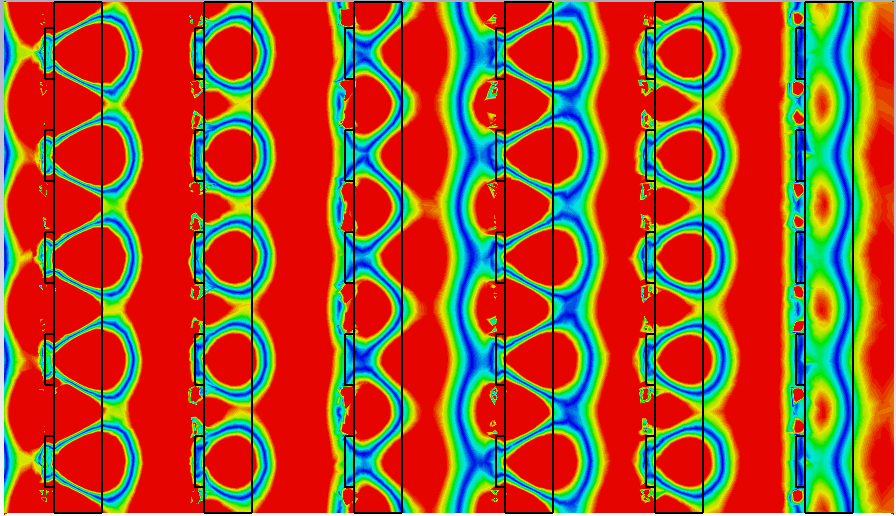


(a)

(b)

(c)

(d)

**Figure S7:** E-field distribution in two conditions. (a) and (b) are in 0-degree phase and 1.06 e + 07 v/m intensity, whereas, (c) and (d) are taken in 90-degree phase and 1.06 e + 07 v/m intensity.

**Software verification**

The tunnel model is designed in HFSS software for verification and it also contains 5×5 array as like as, the E-I shape tunnel designed in CST software. Moreover, the designed tunnel structure creates 11 electron clouds in its first array plat that is exhibits in Figure S8 (a). After that, when the e-filed intensity is increased then it shows its response with 11010111000 binary patterns at 537 THz where the tunnel structure exhibits stop bands characteristics.

z

x

y

**0**

**0**

**0**

**1**

**1**

**1**

**0**

**1**

**0**

**1**

1


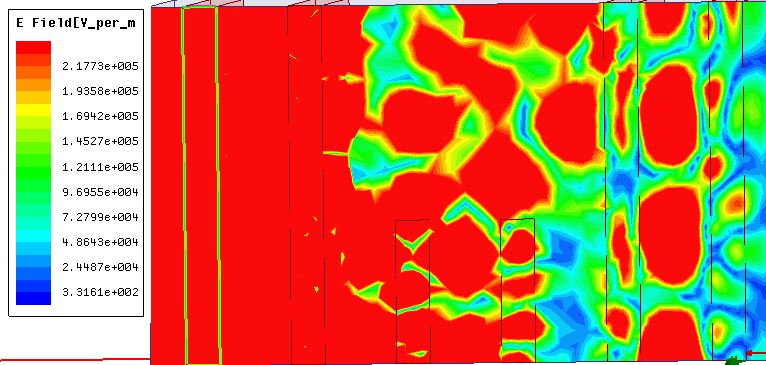

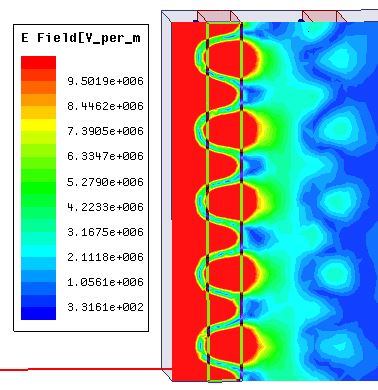


**(a)**

**(b)**

**Figure S8**: HFSS software-based e-field pattern for (a) first two array plate that are shown 11 electron clouds and (b) tunnel responses as 11010111000 patterns in 537 THz (stop band frequency).

**References:**

[S1] Chen, X., Grzegorczyk, T.M., Wu, B., Pacheco, J., Kong, J.A. Roubst method to retrieve the constitutive effective parameters of metamaterials. Physical Review E., 70, 016608, (2004).
